# A Latent Profile Analysis of COVID-19 Trusted Sources of Information among Racial and Ethnic Minorities in South Florida

**DOI:** 10.3390/vaccines10040545

**Published:** 2022-04-01

**Authors:** Robbert J. Langwerden, Eric F. Wagner, Michelle M. Hospital, Staci L. Morris, Victor Cueto, Olveen Carrasquillo, Sara C. Charles, Katherine R. Perez, María Eugenia Contreras-Pérez, Adriana L. Campa

**Affiliations:** 1 Community-Based Research Institute, Florida International University, Miami, FL 33199, USA; wagnere@fiu.edu (E.F.W.); hospitam@fiu.edu (M.M.H.); morrisl@fiu.edu (S.L.M.); sacharle@fiu.edu (S.C.C.); kaperez@fiu.edu (K.R.P.); marcontr@fiu.edu (M.E.C.-P.); 2 Research Center in a Minority Institution, Florida International University, Miami, FL 33199, USA; campaa@fiu.edu; 3 Robert Stempel School of Public Health & Social Work, Florida International University, Miami, FL 33199, USA; 4 Department of Biostatistics, Florida International University, Miami, FL 33199, USA; 5 Department of Medicine, Miller School of Medicine, University of Miami, Miami, FL 33136, USA; vcueto@miami.edu (V.C.); ocarrasquillo@med.miami.edu (O.C.); 6 Department of Dietetics and Nutrition, Florida International University, Miami, FL 33199, USA

**Keywords:** COVID-19, latent class analysis, information sources, trust, vaccines, vaccine hesitancy

## Abstract

By the spring of 2021, most of the adult U.S. population became eligible to receive a COVID-19 vaccine. Yet, by the summer of 2021, the vaccination rate stagnated. Given the immense impact COVID-19 has had on society and individuals, and the surge of new variant strains of the virus, it remains urgent to better understand barriers to vaccination, including the impact of variations in trusted sources of COVID-19 information. The goal of the present study was to conduct a cross-sectional, community-engaged, and person-centered study of trusted sources of COVID-19 information using latent profile analysis (LPA). The aims were to (1) identify the number and nature of profiles of trusted sources of COVID-19 information, and (2) determine whether the trust profiles were predictive of COVID-19 vaccination attitudes and various demographic categories. Participants included mostly racial and ethnic minority individuals (82.4%) recruited by various community-based agencies in South Florida. The LPA evidenced an optimal 3-class solution characterized by low (*n* = 80)-, medium (*n* = 147)-, and high (*n* = 52)-trust profiles, with high trust statistically significantly predictive of vaccination willingness. The profiles identified could be important targets for public health dissemination efforts to reduce vaccine hesitancy and increase COVID-19 vaccination uptake. The general level of trust in COVID-19 information sources was found to be an important factor in predicting COVID-19 vaccination willingness.

## 1. Introduction

A national poll conducted in 2020—at which time no vaccines were available to the public—estimated that the adult COVID-19 vaccine hesitancy level in the United States was 27% [1]. In the fall of 2021, by which time all adults were eligible for vaccination and vaccines were widely available, 31% of U.S. adults had not been vaccinated against COVID-19 [2], and the herd immunity threshold (i.e., 70–90% immunity) had not been reached [3,4]. Vaccination remains extremely important, particularly considering COVID-19 strain variants and additional waves of infection, hospitalization, and morbidity. COVID-19 vaccine hesitancy can be defined as a “delay in acceptance or refusal of vaccination despite availability of vaccination services [4].” Vaccine hesitancy is not a new phenomenon [4,5], and challenges remain in overcoming collective hesitancy to public health vaccination programs of any kind. One of the major challenges is the disproportionate impact COVID-19 has had on underserviced groups in the U.S. The pandemic has exacerbated a wide range of existing racial and ethnic health disparities [6], including vaccination access and uptake [7]. In Florida in particular, COVID-19 health disparities have been identified regarding disproportionate COVID-19 infection rates and increased mortality [8].

The psychological phenomenon of vaccine hesitancy can be explained by several theoretical frameworks. The Theory of Rationed Action (TRA) proposes that willful behavior is a combination of individual factors (attitudes) and social influence (norms) [9]. The more comprehensive Theory of Planned Behavior (TPB) in turn proposes that people’s intentions (i.e., attitudes, perceived control, subjective norms) determine their behavior (i.e., getting vaccinated for COVID-19) across situations [10]. From this perspective, current attitudes predict future individual health behavior. In the context of COVID-19, three TPB factors (attitudes, norms, perceived control) were found to be predictive of willingness to socially distance three months after assessment [11]. Finally, the Health Belief Model (HBM) [12] emphasizes (1) perceived susceptibility to a certain condition, (2) perceived seriousness of the condition, (3) perceived benefits versus barriers of active health behavior, and (4) perceived confidence in being able to take action [12,13]. Furthermore, HBM describes cues to action, such as media coverage, as important factors that could nudge people into action [13]. The TPB, TRA, and HBM models provide guidance on categorizing predictors of behaviors around COVID-19 vaccination.

Against the backdrop of these theoretical frameworks, a range of recently conducted empirical studies have identified predictors of COVID-19 vaccine hesitancy. Predictive factors of hesitancy can be categorized as socio-demographic predictors [14,15,16], barriers (including perceived susceptibility to COVID-19 and perceived COVID-19 severity) [17,18], and motivators [19]. In line with the HBM, the relationship between the attitudinal factors (perceived susceptibility, perceived barriers and motivators, perceived control) and vaccination intention was strongest in the U.S. compared to several other countries [20]. The HBM was found to predict COVID-19 vaccination intentions in populations in both the U.S. [20,21,22,23] and several European countries [24,25], yet additional research is needed to further investigate the role of various COVID-19 vaccination predictors.

A person-centered approach to studying these factors can contribute to the existing literature by investigating sets of factors that influence vaccination attitudes and uptake. For example, a large convenience-sampling-based study in the U.S. indicated four classes to be predictive of COVID-19 hesitancy—“development concerns, ” “pro-vaccine,” “unsure/hesitant,” and “anti-vaccine [26].” Another study found three out of six major vaccine hesitancy classes, all predictive of COVID-19 vaccination status and intention, including (1) a “single-dose preference group,” (2) a “two-dose preference group,” and (3) a “vaccinate only once group [27].” As predicted by the HBM, it is not only profiles of perceived barriers that are important, but also the acknowledgement that public health campaigns can harness important motivators of vaccination through trusted channels of information. Various trusted sources, such as a general “trust in science,” was found to distinguish between vaccine misinformation belief profiles in a person-centered study [28]. Further person-centered research of trusted sources of information, and the interactions among these sources across different individuals, communities, and geographic regions, maps well onto the HBM. Previous studies, however, have not always focused on the differences among racial and ethnic minority populations, which is needed given the disproportionate impact COVID-19 has had on these populations.

Informed by the theoretical frameworks and research in the field, this study aimed to assess predictive profiles associated with vaccine hesitancy in a metropolitan region in South Florida. This study contributes to the literature by identifying profiles that may be predictive of vaccination status and could therefore be useful in increasing uptake. We took a community-engaged, person-centered approach to investigate profiles of trusted sources of COVID-19 information among racial and ethnic minority community members in the southeastern United States. This study population is unique because it utilized community sampling in partnership with community-based organizations (CBO), which serve urban and underserved communities in a demographically unique and diverse region of the United States. Data were collected in line with the goals of the Community Engaged Alliance against COVID-19 disparities initiative (CEAL) [29]. The current study’s aims were to (1) assess the optimal number and nature of profiles of trusted sources of COVID-19 information, and (2) investigate whether the established profiles have discriminant validity regarding vaccine willingness, vaccination status, likelihood of getting vaccinated, and demographic covariates. If certain profiles are determined to be more common among the unvaccinated, these sets of information sources can be leveraged, particularly in underserved communities. We predicted that there will be lower trust of all information sources among the unvaccinated, particularly trust in those from the established medical science community. However, our general analytic approach was exploratory in examining associations between profiles and vaccination willingness.

## 2. Materials and Methods

Participants were recruited by two collaborative FL-CEAL research groups from Florida International University (FIU) and the University of Miami (UM). The FIU researchers (Site 1) recruited participants though five partnering CBOs in the South Florida larger metropolitan area (Miami–Dade, Broward, and Palm Beach Counties). The UM researchers (Site 2) recruited participants though various community organizations in the overlapping areas (Miami–Dade and Broward Counties), including through school-based organizations, youth sports, health care organizations, as well as community assistance organizations (i.e., food pantries). Study surveys were administered via an online survey link through Research Electronic Data Capture (REDCap; [30]). Participants were referred to the survey by community organizations by clicking on a link, after which they were shown an informational letter (providing anonymous consent), completed the survey, and in a separate data collection tool filled out their contact information for distribution of gift cards. Each site gave out differently valued gift cards (FIU $10, UM $25). All surveys were collected between May and July 2021, at which time all adults (18 years or older) were eligible to receive a COVID-19 vaccine in the State of Florida. Due to technological difficulties at Site 1 (i.e., a bot attempting to complete surveys), active data collection was discontinued mid-May 2021. At Site 2, data collection was discontinued in July 2021 due to data saturation. The online REDCap CEAL Common survey was developed and translated by the Steering Committee and Evaluation Work Group of the Florida Community Engagement Alliance against COVID-19 Disparities (CEAL; [29,31]) and was adapted to consist of 28 questions about (1) COVID-19 prevention behaviors, (2) testing behaviors, (3) intentions to get a COVID-19 vaccine, (4) trusted sources of information about COVID-19, (5) COVID-19 clinical trials, clinical trial registration, and enrollment behaviors, (6) self-reported awareness and knowledge about COVID-19 clinical trials, (7) willingness and intentions to register or enroll in COVID-19 clinical trials, (8) trust regarding COVID-19 clinical trials, and (9) social determinants of health and demographics. The items had a reading level of 7.2 (Flesch–Kincaid) and were designed for electronic administration. The survey instrument was available in English, Spanish, and Haitian-Creole, all of which were translated by certified medical translators.

Regarding the current study, we focused our analyses on trust in 11 different sources of information, which were was assessed on a three-point scale (1, *not at all*, 2, *a little*, 3, *a great deal*) including a *don’t know* option, on a select-all-that-apply basis with the item “*How much do you trust each of these sources to provide correct information about COVID-19*?” These 11 information sources included (1) your doctor or health care provider, (2) your faith leader, (3) your close friends and members of your family, (4) people you go to work or class with or other people you know, (5) news on the radio, TV, online, or in newspapers, (6) your contacts on social media, (7) the U.S. Government, (8) the U.S. Coronavirus Task Force, (9) leaders in your community, (10) local politicians, and (11) billboards. Participants with all missing data or “don’t know” answer responses were excluded from analysis and all other participants were included. Further, we assessed the social determinants of health and accompanying demographic disparities on COVID-19 vaccinations and willingness as outcomes. In addition, participants were asked whether they had received the COVID-19 vaccine, and if not, how likely they were to take an approved COVID-19 vaccine (“*How likely are you to get an approved COVID-19 vaccine when it becomes available?*”; 1, *not likely at all*, to 7, *very likely*). Vaccination status (yes/no) and likelihood of taking the vaccine were post hoc combined into a “vaccination willingness spectrum” variable (1, *not likely at all*, to 8, *being vaccinated*). Mplus version 8 [32] was used to conduct independent latent profile analyses of 11 continuous variables of trust in COVID-19 information sources, using maximum likelihood parameter estimates (MLR). Once the optimal number of classes (i.e., profiles) was established, we used the class probabilities outputted to regress vaccination willingness and other outcomes onto the profiles. The Mplus CPROB output function was used for this step in the analysis. We conducted multiple iterations of latent profile analyses to detect the best-fitting model and therefore optimal number of profiles. We inputted the 11 trusted resources of information as continuous predictors in the model, such that Mplus calculated the fit based on 1, 2, 3, and 4 classes.

## 3. Results

### 3.1. Participant Demographics

Analyses were conducted on the combined sample (*N* = 279) after removing participants who either had many missing responses (*n* = 18) on the trusted sources variables or who were younger than 18 (*n* = 1) at the time of survey completion. The sample’s demographics are summarized in Table 1. Site 1 included 152 individuals and Site 2 included 127 individuals. Comparing Site 1 to Site 2, there were statistically significant differences in vaccination status, identifying as Black or African American, working a full-time job, and working without pay. There was no significant difference between the samples on vaccination willingness (combined vaccination status and likelihood), despite there being a significant difference between vaccination status (yes/no) only. We did not control for these variables, since both sites recruited through a variety of community organizations and represented the diversity of South Florida. We included Miami–Dade County census data in Table 1 to contextualize the representation of South Florida’s communities. Out of the 279 participants, 230 participants (82.4%) identified as underrepresented minority based on race or ethnicity.

### 3.2. Latent Profile Analysis

**Figure 1:**
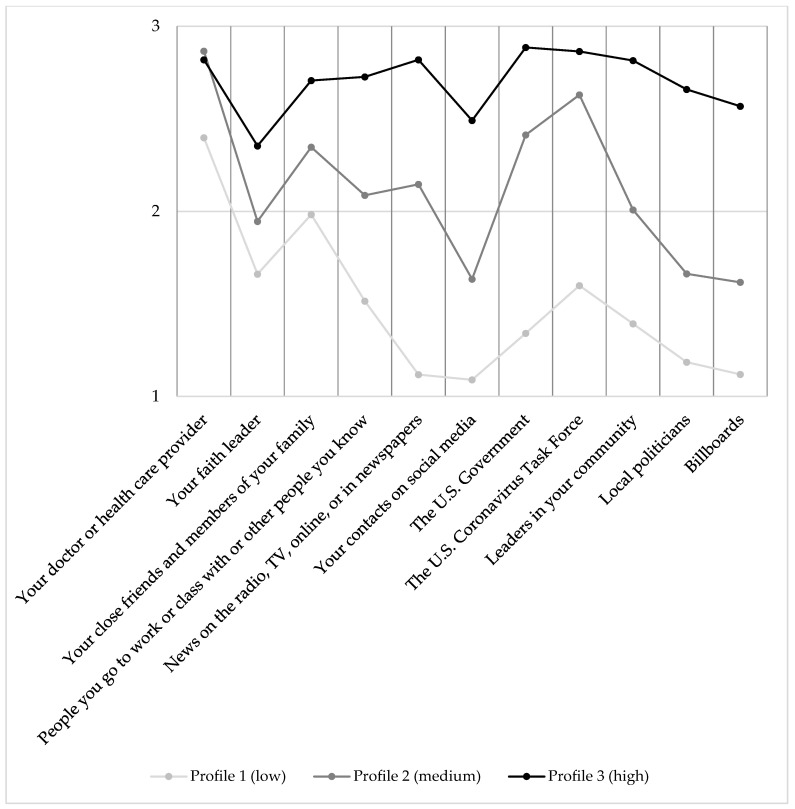
Visual display of LPA results of the 3-class solution of 11 trusted sources of information. Note: Profile 1 (*n* = 80), Profile 2 (*n* = 147), Profile 3 (*n* = 52).

**Figure 2:**
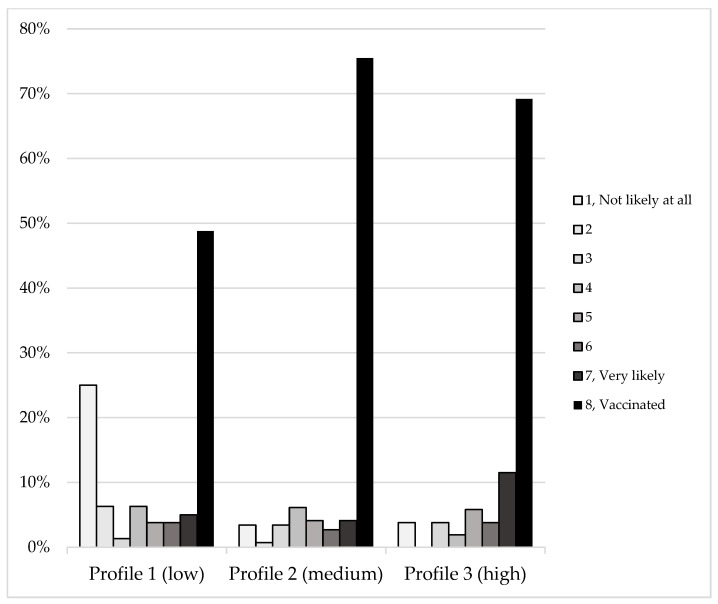
Vaccination willingness scores as proportions (%) by trust profile. Note: All percentages use the profile group’s size as denominator; Profile 1 (*n* = 80), Profile 2 (*n* = 147), Profile 3 (*n* = 52).

The LPA output indicated that the 3-class solution was superior to the 1-class and 2-class solution indicators. The 3-class was also a better solution than the 4-class, which did not converge, indicating a poor model fit. The 3-class solution produced the most optimal class-fit indicators (AIC = 4997.82, BIC = 5164.85, ABIC = 5018.99, LRT *p* < 0.001, entropy = 0.888), indicating that 88.8 percent of the sample could be reliably classified in one of the three classes. We repeated the 3-class solutions for various ethnic and racial subsamples, including only Hispanic participants, only non-Hispanic participants, Black or African American participants, non-Black or non-African American participants, white participants, and non-white participants. We inspected the visual output of each of these models, which did not yield any major differences and the model was therefore regarded as robust and generalizable. Regarding the main 3-class solution, three distinct profiles of trusted information sources emerged, which we labelled as low trust (*n* = 80), medium trust (*n* = 147), and high trust (*n* = 52). The results are graphicly displayed in Figure 1 and Figure 2.

Among all groups, “your doctor or health care provider” was trusted highly, relative to other sources. This information source is an exception in the sense that the medium-trust group trusts this source more highly than the high-trust group, although both are comparably high. Second, “your faith leader” was trusted relatively highly compared to other sources, and this was particularly true for the low and medium groups. Further, trust in “your close friends and members of your family” was also relatively high in all groups. “Coworkers, fellow students, or other acquaintances” were trusted a medium amount in all groups, similar to “news (on radio, tv, online, or in newspapers).” Compared to trusted sources, between and within each profile, this was a relatively low-trusted source. “The U.S. Government” and “U.S. Coronavirus Task Force” both were trusted relatively highly within each profile. However, the low-trust profile did not trust these two sources as much as the medium- and high-trust groups. “Leaders in your community” were trusted relatively low within each profile, followed by “local politicians” and “billboards,” both of which were trusted very lowly as well. Comparing the three profiles, the same rough trust pattern exists with regard to trust in individual sources. The results indicate that the overall level of trust is indicative of the profile, rather than trust in one or more specific individual sources.

### 3.3. Regression Analysis and Inferential Statistics

First, Chi-square tests for independence indicated the profiles differentiated between vaccination status (yes/no), which was statistically significant (χ^2^ = 16.88, *p* < 0.001). Interestingly, the results indicated that profile 2 (medium trust) had the highest vaccination rate (75.5%), followed by profile 3 (high trust; 69.2%) and profile 1 (low trust; 48.8%). Second, using the Mplus outputted class probabilities (i.e., the most likely profile each participant is classified into), we conducted linear regression analyses using the profile as a predictor of vaccination willingness (vaccination status and likelihood combined). Univariate linear regression was applied to regress vaccine willingness onto profile membership among the entire sample (*N* = 279). The regression model was statistically significant (*R^2^* = 0.08, *F*(1,278) = 23.9, *p* < 0.001), and we found that profile membership significantly predicted vaccine willingness (*β* = 0.986, *p* < 0.001), and with every 1 unit increase in profile (low to medium to high), the vaccine willingness increases by 0.986. This model yields a medium effect size. Lastly, among the 93 unvaccinated participants, univariate linear regression indicated that there was a statistically significant effect of profiles predicting vaccine likelihood (*R^2^* = 0.16, *F*(1,91) = 17.3, *p* < 0.001), such that for every 1 unit increase in profile, the vaccine likelihood increases by 1.21 (*p* < 0.001). We tested whether profile membership differs by demographic outcome using Chi-square tests and ANOVA(see Table 2). Two outcomes were found to be statistically significant. First, a statistically significant difference was found between sites, such that the higher the profile (i.e., general trust), the higher the proportion of Site 1 (FIU) individuals. Second, regarding gender, for which a statistically significant effect was found, female participants were more often classified as Profile 1 (low trust; 33.8%) and Profile 3 (high trust; 42%) compared to Profile 2 (medium trust; 23.8%). Nonbinary, genderqueer, and genderfluid only were always classified as Profile 3 and never as Profile 1 or 2.

## 4. Discussion

We successfully administered close to 300 surveys across two sites in a diverse metropolitan region in South Florida, of which our sample was relatively representative. We conducted a community-based, COVID-19 trusted sources of information profile study, using a sophisticated person-centered statistical approach; latent profile analysis. The results showed that a 3-class model was optimal for characterizing variation in trusted sources of COVID-19 information among our participants. We found that profile membership predicted whether one is likely to get or be vaccinated but was not associated with any other measured demographic variable, except for gender. We conclude that the overall level of trust in COVID-19 information sources, rather than trust in a specific source of information, is the most important factor in predicting COVID-19 vaccination willingness among this group of majority racial and ethnic minority individuals.

Profile 1, which we labeled as the low-trust group, is characterized by low trust across all information sources, except for trust in doctors and health care providers, friends and family, and the U.S. Coronavirus Task Force. The relatively high trust in medical and scientific authorities is in contrast with the generally low trust in all other entities. Profile 2, which we labeled as the medium-trust group, has a generally medium-to-high trust in all information sources, apart from relatively lower trust in social media, religious leaders, and billboards, as well as relatively higher trust in doctors and health care providers, the U.S. Government, and the U.S. Coronavirus Task Force. Profile 3, labeled as the high-trust group, is the smallest group of the three and is characterized by generally high-to-very-high trust in all information sources. The few minor exceptions to Profile 3 are religious leaders, social media, and billboards (both relatively lower trust). It is worth noting that Profile 2 and 3 separate themselves from Profile 1 by a generally higher trust in the U.S. Coronavirus Task Force. Importantly, we found that these profiles, and whether participants belong to these groups, were predictive of whether one is likely to get or be vaccinated, and this effect is statistically significant and medium in size. We did find other statistically significant discriminators between the profiles, including gender. However, due to the small sample size for the non-binary, genderfluid, or genderqueer group, meaningful conclusions cannot be drawn.

The three identified profiles may have important implications for COVID-19 and COVID-19 vaccine information distribution among racial and ethnic minority individuals. First, when inspecting the commonalities between the groups, one could conclude that all three groups have relatively high trust in doctors and health care providers and the U.S. Coronavirus Task Force, suggesting a general high trust in medical and public health professionals and indicating that this may be utilized as an important outlet for COVID-19 vaccine information across the general population. Encouragement of COVID-19 vaccination and prevention behaviors by medical providers may be an effective tool for addressing vaccine hesitancy. While many people cancelled or delayed their in-person medical appointments, there may be remote or digital avenues through which medical experts could expand their reach. All three groups also were characterized by relatively high trust in their friends and family for COVID-19 information. This finding emphasizes the role of social norms [35] and is in line with the Theory of Reasoned Action [9] and Theory of Planned Behavior [10]. However, it does not tell us whether friends and family are facilitators of hesitancy, as it is likely that friends and family share the same attitudes and approaches to the COVID-19 pandemic. It is also unclear whether those attitudes are characterized by a hesitancy toward COVID-19 vaccines or not. Interestingly, “the news” as a general COVID-19 information source was trusted relatively highly in the medium- and high-trust groups, but less so in the low-trust groups. An important caveat here is that we did not specify any specific news sources, which would serve as a qualifier in this context. Finally, we want to emphasize that there are several information sources that show a relatively lower level of trust across all three profiles, including billboards and social media contacts, which are likely to be ineffective COVID-19 vaccine information channels.

A few limitations need to be considered. First, several items in the survey (i.e., “the news” and “local politicians”) were formulated rather generically, such that they might be interpreted differently by different individuals. Second, we found statistically significant differences between the two samples on several demographic variables, including gender. Our results merit cautious interpretation, as women disproportionately constitute most of the Floridian vaccinated group [36]. More generally, our results may only be representative of this specific region and population of the United States and not generalizable to other states or countries. However, the diverse minority–majority and largely foreign-born population of the region is representative of a multitude of countries and in some ways of other urban regions in the United States.

Future research could focus on identifying and studying how these trusted sources can be leveraged to promote vaccinate uptake among vaccine-hesitant and unvaccinated individuals. A strength of the present study is that it was community-based and community-partnered; we recommend that future studies maximize the validity of their results by adopting a similar approach to recruiting community members.

## 5. Conclusions

Using a person-centered exploration of trust in COVID-19 information sources, we identified three profiles among majority racial and ethnic minority individuals, each representing a different level of overall trust in 11 information sources. These profiles predicted the vaccination likelihood in a large, diverse sample representative of South Florida.

## Figures and Tables

**Table 1:** Demographic Characteristics of Survey Respondents: overall, by source, and compared to census rates.

Characteristic	Site 1	Site 2	Combined	Miami–Dade County
	(*n* = 152)	(*n* = 127)	(*N* = 279)	Census ^1^
Vaccinated * ^2^	110 (72.4%)	76 (59.8%)	186 (66.7%)	79%
Not vaccinated	42 (27.6%)	51 (40.2%)	93 (33.3%)	21%
Age	*M* = 40.6	*M* = 40.8	*M* = 40.7	*M* = 40.5
	*SD* = 15.4	*SD* = 14.2	*SD* = 14.8	
	18–82	18–80	18–82	
Gender				
Female	103 (68.2%)	89 (70.6%)	192 (69.3%)	51.42%
Male	47 (31.1%)	36 (28.6%)	83 (30%)	48.58%
Nonbinary, genderqueer, or genderfluid	1 (0.7%)	1 (0.8%)	2 (0.7%)	
Race				
White	104 (68.4%)	77 (60.6%)	181 (64.9%)	75.8%
Black or African American *	30 (19.7%)	40 (31.5%)	70 (25.1%)	16.42%
Asian	8 (5.3%)	3 (2.4%)	11 (3.9%)	1.53%
American Indian or Alaska Native	2 (1.3%)	1 (0.8%)	3 (1.1%)	0.21%
Native Hawaiian or Other Pacific Islander	1 (0.7%)	0 (0%)	1 (0.4%)	0.02%
Prefer not to answer	7 (4.6%)	8 (6.3%)	15 (5.4%)	
Ethnicity				
Hispanic or Latino	102 (68%)	71 (57.7%)	173 (63.4%)	71.51%
Race & Ethnicity				
Hispanic or Latino & Black or African American	8 (5.3%)	2 (1.6%)	10 (3.7%)	
Hispanic or Latino & American Indian or Alaska Native	2 (1.3%)	1 (0.8%)	3 (1.1%)	
Hispanic or Latino & Native Hawaiian or Pacific Islander	1 (0.7%)	0 (0%)	1 (0.4%)	
Sexual Orientation				
Bisexual	7 (4.8%)	1 (0.8%)	8 (3%)	
Gay	7 (4.8%)	2 (1.6%)	9 (3.3%)	
Lesbian	2 (1.4%)	1 (0.8%)	3 (1.1%)	
Straight	127 (87.6%)	120 (95.2%)	247 (91.1%)	
Other	2 (1.4%)	2 (1.6%)	4 (1.5%)	
Born in the U.S.				
Yes	83 (57.6%)	70 (57.4%)	153 (57.5%)	45.4%
English as first language				
No	47 (31.3%)	35 (28.5%)	82 (30%)	77%
Educational level				
Less than high school	0 (0%)	1 (0.8%)	1 (0.4%)	9.39%
Some high school	2 (1.3%)	3 (2.4%)	5 (1.8%)	8.43%
High school graduate or GED	32 (21.3%)	27 (21.6%)	59 (21.5%)	27.31%
Associates or technical degree	28 (18.7%)	21 (16.8%)	49 (17.8%)	9.40%
Bachelor’s degree	54 (36%)	45 (36%)	99 (36%)	19.32%
Graduate degree	34 (22.7%)	28 (22.4%)	62 (22.5%)	8.29%
Employment status				
Working for pay—part time	34 (22.4%)	25 (19.7%)	59 (21.1%)	
Working for pay—full time *	72 (47.4%)	75 (59.1%)	147 (52.7%)	
Working without pay *	4 (2.6%)	0 (0%)	4 (1.4%)	
On leave	0 (0%)	1 (0.8%)	1 (0.4%)	
Unemployed and looking for a job	12 (7.9%)	7 (5.5%)	19 (6.8%)	
Unemployed and not looking for a job	2 (1.3%)	3 (2.4%)	5 (1.8%)	
Retired	5 (3.3%)	4 (3.1%)	9 (3.2%)	
Staying at home, taking care of the home or others	8 (5.3%)	7 (5.5%)	15 (5.4%)	
Not able to work because of disability	1 (0.7%)	0 (0%)	1 (0.4%)	
Going to school	23 (15.1%)	16 (12.6%)	39 (14%)	
Other	4 (2.6%)	2 (1.6%)	6 (2.2%)	

Note: Percentages reflect valid percentages. ^1^ Census data was retrieved from the Miami–Dade County tracker [33]. ^2^ Vaccination data were retrieved from [34]. * Statistically significant difference.

**Table 2:** Test results of profile membership by demographic outcome.

Covariate	Value	*p*	Effect Size
Site	12.39	**0.002**	0.2
Age	1.67	0.2	0.006
U.S. Born	0.82	0.67	0.06
Gender	16.54	**0.002**	0.17
Sexual Orientation	13.51	0.1	0.16
Hispanic/Latino	5.32	0.07	0.14
White	0.533	0.77	0.04
Black or African American	0.68	0.71	0.05
Asian	3.94	0.14	0.12
American Indian or Alaska Native	0.7	0.7	0.05
Native Hawaiian or Pacific Islander	2.5	0.29	0.1
Educational level	2.57	0.99	0.07
Income	8.89	0.84	0.14
Full-time employment	3.31	0.19	0.11

Note: All significant tests were conducted using Pearson Chi-square tests of independence, except for age, for which an ANOVA was conducted. Effect sizes represent Cramer’s V (0–0.3 weak, 0.4–0.5 medium, >0.5 strong) for the Chi-square tests and r-squared for the ANOVA. Bolded *p*-values are significant at the 0.05 level.

## Data Availability

Not applicable.
